# *trans*-4-(2-Amino-5-bromo-6-methyl­pyrimidin-4-ylamino)-1-methyl­cyclo­hexa­nol

**DOI:** 10.1107/S1600536809035533

**Published:** 2009-09-09

**Authors:** Jacqui E. Hoffman, Henry Cheng, Arnold L. Rheingold, Antonio DiPasquale, Alex Yanovsky

**Affiliations:** a Pfizer Global Research and Development, La Jolla Labs, 10770 Science Center Drive, San Diego, CA 92121, USA; b Department of Chemistry and Biochemistry, University of California, San Diego, 9500 Gilman Drive, La Jolla, CA 92093, USA

## Abstract

The title compound, C_12_H_19_BrN_4_O, represents the minor component of the two products obtained in a series of transformations involving the Grignard reaction of *tert*-butoxy­carbonyl-protected 4-amino­cyclo­hexa­none with MeMgBr, and subsequent inter­action of the obtained amino-substituted cyclo­hexa­nol with 4-chloro-6-methyl­pyrimidin-2-amine followed by bromination with *N*-bromo­succinimide. The X-ray structure showed that this product represents a *trans* isomer with respect to the amino and hydr­oxy substituents in the cyclo­hexyl ring; the dihedral angle between the amino­pyrimidine plane and the (noncrystallographic) mirror plane of the substituted cyclo­hexyl fragment is 33.6 (3)°. Only two of the four potentially ‘active’ H atoms participate in inter­molecular N—H⋯O and O—H⋯N hydrogen bonds, linking the mol­ecules into layers parallel to the (10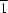) plane.

## Related literature

For the structure of a similar *N*-pyrimidine derivative of amino­cyclo­hexane, see Melguizo *et al.* (2003 ▶).
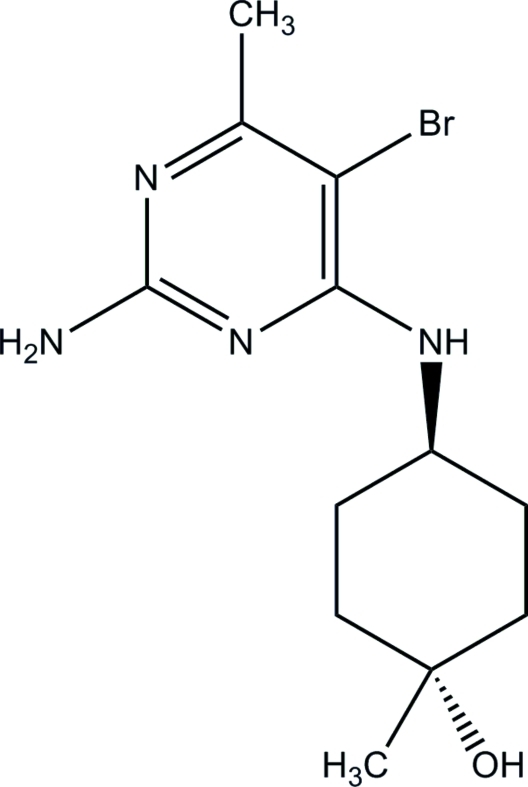


## Experimental

### 

#### Crystal data

C_12_H_19_BrN_4_O
*M* *_r_* = 315.22
Monoclinic, 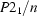

*a* = 9.9514 (18) Å
*b* = 7.1879 (11) Å
*c* = 19.566 (4) Å
β = 91.053 (3)°
*V* = 1399.3 (4) Å^3^
*Z* = 4
Mo *K*α radiation
μ = 2.93 mm^−1^
*T* = 198 K
0.10 × 0.10 × 0.08 mm

#### Data collection

Siemens P4 with APEX CCD area-detector diffractometer
Absorption correction: multi-scan (*SADABS*; Bruker, 2001 ▶) *T*_min_ = 0.758, *T*_max_ = 0.799
8853 measured reflections
3251 independent reflections
2500 reflections with *I* > 2σ(*I*)
*R*_int_ = 0.030

#### Refinement

*R*[*F* ^2^ > 2σ(*F* ^2^)] = 0.041
*wR*(*F* ^2^) = 0.114
*S* = 1.05
3251 reflections
166 parameters
H-atom parameters constrained
Δρ_max_ = 0.83 e Å^−3^
Δρ_min_ = −0.77 e Å^−3^

### 

Data collection: *SMART* (Bruker, 1997 ▶); cell refinement: *SAINT* (Bruker, 1997 ▶); data reduction: *SAINT*; program(s) used to solve structure: *SIR2004* (Burla *et al.*, 2005 ▶); program(s) used to refine structure: *SHELXL97* (Sheldrick, 2008 ▶); molecular graphics: *ORTEP-32* (Farrugia, 1997 ▶); software used to prepare material for publication: *WinGX* (Farrugia, 1999 ▶).

## Supplementary Material

Crystal structure: contains datablocks global, I. DOI: 10.1107/S1600536809035533/bg2293sup1.cif

Structure factors: contains datablocks I. DOI: 10.1107/S1600536809035533/bg2293Isup2.hkl

Additional supplementary materials:  crystallographic information; 3D view; checkCIF report

## Figures and Tables

**Table 1:** Hydrogen-bond geometry (Å, °)

*D*—H⋯*A*	*D*—H	H⋯*A*	*D*⋯*A*	*D*—H⋯*A*
N3—H3*A*⋯O1^i^	0.88	2.03	2.828 (3)	151
O1—H1⋯N1^ii^	0.84	2.02	2.803 (3)	155

Symmetry codes: (i) 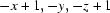; (ii) 
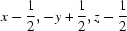
.

**Fig. 1.**
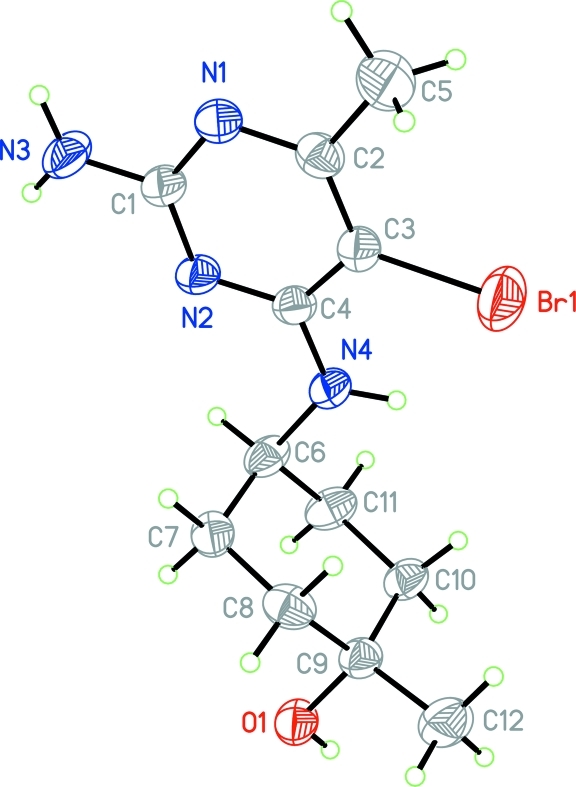
Molecular structure of the title compound showing 50% probability displacement ellipsoids and atom numbering scheme; H atoms are drawn as circles with arbitrary small radius.

**Fig. 2.**
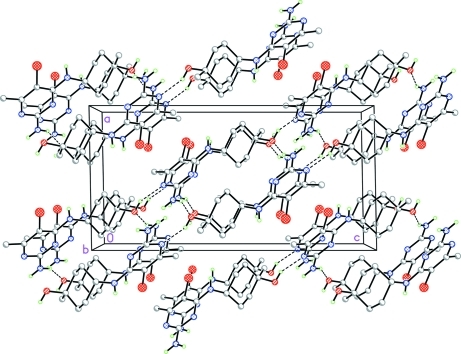
Packing diagram for the title compound viewed approximately along the b axis. H-Bonds are shown as dashed lines; H atoms bound to carbon atoms are omitted.

## supplementary crystallographic information

### Comment

The Grignard reaction of *tert*-butoxycarbonyl(BOC)-protected 4-aminocyclohexanone, 4-C_4_H_9_OC(O)N(H)C_6_H_9_O, with MeMgBr produced the mixture of *cis*- and *trans*- 4-BOC-amino-1-methyl-cyclohexanols, which was subsequently reacted with 4-chloro-6-methylpyrimidin-2-amine and then brominated with *N*-bromosuccinimide. The isomeric mixture of the products was separated by means of flash chromatography and the corresponding X-ray structural study of the minor isomer showed that the title compound represents a *trans*-isomer with respect to the amino and hydroxy substituents in the cyclohexane ring (Fig. 1). The plane of the diaminopyrimidine C1/C2/C3/C4/N1/N2/N4 fragment forms a dihedral angle of 33.6 (3)° with the approximate mirror plane of the cyclohexyl fragment, namely the plane passing through N4/C6/C9/C12/O1. This conformation is significantly different from that observed in the related compound described in Melguizo *et al.*, 2003, where the dihedral angle formed by the aminopyrimidine plane and the mirror plane of the cyclohexyl ring is just 13.6°.

There are four H atoms in the molecule, which are capable of H-bond formation. However, only two of them (H1 and H3A) participate in intermolecular H-bonds (Table 1), which link the molecules into layers parallel to the (1,0,-1) plane of the crystal (Fig. 2).

### Experimental

Synthesis of *tert*-butyl 4-hydroxy-4-methylcyclohexylcarbamate. To a cooled (0°C) solution of 4-*N*-boc-amino-cyclohexanone (4.79 g, 22.5 mmol) in tetrahydrofuran (190 ml) was added methylmagnesium bromide (3 *M* solution in diethyl ether, 22.5 ml, 67.2 mmol). The ice bath was removed and the reaction was stirred at room temperature for 6 h, and then quenched with saturated ammonia chloride and water. The reaction mixture was concentrated and residue was dissolved in ethyl acetate and washed with saturated ammonia chloride, dried (MgSO4), filtered, and concentrated again. The crude product was purified by flash chromatography eluting with hexanes/ethyl acetate (10–50%) then chloroform/methanol (10%) to afford *tert*-butyl 4-hydroxy-4-methylcyclohexylcarbamate as a mixture of isomers (2.72 g, 53%).

Synthesis of 4-(2-amino-6-methylpyrimidin-4-ylamino)-1-methylcyclohexanol. To a cooled (0°C) solution of *tert*-butyl 4-hydroxy-4-methylcyclohexylcarbamate (2.72 g, 11.9 mmol) in dichloromethane was added hydrochloric acid (2 *M* solution in diethyl ether, 10 eq). The ice bath was removed and the solution was stirred at room temperature for 6 hrs then concentrated to afford 4-amino-1-methylcyclohexanol hydrochloride, which was used without further purification. A solution of 2-amino-4-chloro-6-methylpyrimidine (2.20 g, 15.4 mmol), 4-amino-1-methylcyclohexanol hydrochloride, and diisopropylethyl amine (7.6 ml, 44 mmol) in dimethyl acetamide (52 ml) was heated to 160°C in a sealed tube overnight. The reaction mixture was concentrated, the solids were slurried in chloroform and the filtrate was concentrated again. The crude product was purified by flash choromatagraphy eluting with chloroform/7 N ammonia in methanol followed by SFC chromatography to afford 4-(2-amino-6-methylpyrimidin-4-ylamino)-1-methylcyclohexanol as a mixture of isomers (600 mg, 17% over 2 steps).

Synthesis of 4-(2-amino-5-bromo-6-methylpyrimidin-4-ylamino) -1-methylcyclohexanol. To a solution of 4-(2-amino-6-methylpyrimidin-4-ylamino)-1-methylcyclohexanol (600 mg, 2.54 mmol) in dichloromethane (20.0 ml) was added *N*-bromosuccinimide (452 mg, 2.54 mmol). After stirring for 2.5 hrs at room temperature, the solution was concentrated. The residue was dissolved in ethyl acetate (450 ml) and washed with 50% saturated sodium carbonate, brine, dried (MgSO4), filtered, and concentrated again. The crude product was purified by flash chromatography to afford major (407 mg, 51%) and minor (151 mg, 19%) isomers of 4-(2-amino-5-bromo-6-methylpyrimidin-4-ylamino)-1-methylcyclohexanol. Minor isomer (the title compound) was subjected to the X-ray study and proved to be the *trans*-isomer.

1H NMR spectra for major (*cis*) isomer: (400 MHz, DMSO-d6) δp.p.m. 1.11 (s, 3 H) 1.26 - 1.37 (m, 2 H) 1.52 - 1.61 (m, 4 H) 1.61 - 1.73 (m, 2 H) 2.17 (s, 3 H) 3.77 - 3.87 (m, 1 H) 4.06 (s, 1 H) 5.73 (d, J=8.34 Hz, 1 H) 6.08 (s, 2 H)

1H NMR spectra for minor (*trans*) isomer (the title compound): (400 MHz, DMSO-d6) δp.p.m. 1.16 (s, 3 H) 1.36 - 1.45 (m, 2 H) 1.45 - 1.56 (m, 4 H) 1.64 - 1.73 (m, 2 H) 2.17 (s, 3 H) 3.87 - 3.97 (m, 1 H) 4.28 (s, 1 H) 5.93 (d, J=8.59 Hz, 1 H) 6.11 (s, 2 H)

### Refinement

All H atoms were placed in geometrically calculated positions (O—H 0.84 Å, N—H 0.88 Å, C—H 0.98 Å, 0.99 Å, 1.00 Å for methyl, methylene and methyne H atoms respectively) and included in the refinement in riding motion approximation. The *U*_iso_(H) were set to 1.2*U*_eq_ of the carrying atom for methylene, methyne and amine groups, and 1.5*U*_eq_ for methyl and hydroxyl H atoms.

### Crystal data

**Table:**

C_12_H_19_BrN_4_O	*F*(000) = 648
*M**_r_* = 315.22	*D*_x_ = 1.496 Mg m^−^^3^
Monoclinic, *P*2_1_/*n*	Mo *K*α radiation, λ = 0.71073 Å
Hall symbol: -P 2yn	Cell parameters from 2554 reflections
*a* = 9.9514 (18) Å	θ = 2.3–26.0°
*b* = 7.1879 (11) Å	µ = 2.93 mm^−^^1^
*c* = 19.566 (4) Å	*T* = 198 K
β = 91.053 (3)°	Prism, colorless
*V* = 1399.3 (4) Å^3^	0.10 × 0.10 × 0.08 mm
*Z* = 4	

### Data collection

**Table:**

Siemens P4 with APEX CCD area-detector diffractometer	3251 independent reflections
Radiation source: fine-focus sealed tube	2500 reflections with *I* > 2σ(*I*)
graphite	*R*_int_ = 0.030
φ and ω scans	θ_max_ = 28.3°, θ_min_ = 2.1°
Absorption correction: multi-scan (*SADABS*; Bruker, 2001)	*h* = −13→13
*T*_min_ = 0.758, *T*_max_ = 0.799	*k* = −9→3
8853 measured reflections	*l* = −25→25

### Refinement

**Table:**

Refinement on *F*^2^	Primary atom site location: structure-invariant direct methods
Least-squares matrix: full	Secondary atom site location: difference Fourier map
*R*[*F*^2^ > 2σ(*F*^2^)] = 0.041	Hydrogen site location: inferred from neighbouring sites
*wR*(*F*^2^) = 0.114	H-atom parameters constrained
*S* = 1.05	*w* = 1/[σ^2^(*F*_o_^2^) + (0.0567*P*)^2^ + 0.5508*P*] where *P* = (*F*_o_^2^ + 2*F*_c_^2^)/3
3251 reflections	(Δ/σ)_max_ < 0.001
166 parameters	Δρ_max_ = 0.83 e Å^−^^3^
0 restraints	Δρ_min_ = −0.77 e Å^−^^3^

### Special details

**Table:**

Geometry. All e.s.d.'s (except the e.s.d. in the dihedral angle between two l.s. planes) are estimated using the full covariance matrix. The cell e.s.d.'s are taken into account individually in the estimation of e.s.d.'s in distances, angles and torsion angles; correlations between e.s.d.'s in cell parameters are only used when they are defined by crystal symmetry. An approximate (isotropic) treatment of cell e.s.d.'s is used for estimating e.s.d.'s involving l.s. planes.
Refinement. Refinement of *F*^2^ against ALL reflections. The weighted *R*-factor *wR* and goodness of fit *S* are based on *F*^2^, conventional *R*-factors *R* are based on *F*, with *F* set to zero for negative *F*^2^. The threshold expression of *F*^2^ > σ(*F*^2^) is used only for calculating *R*-factors(gt) *etc*. and is not relevant to the choice of reflections for refinement. *R*-factors based on *F*^2^ are statistically about twice as large as those based on *F*, and *R*- factors based on ALL data will be even larger.

### Fractional atomic coordinates and isotropic or equivalent isotropic displacement parameters (Å^2^)

**Table:**

	*x*	*y*	*z*	*U*_iso_*/*U*_eq_	
C1	0.5559 (2)	0.1796 (4)	0.70305 (12)	0.0340 (5)	
C2	0.4564 (2)	0.4484 (3)	0.74028 (12)	0.0345 (5)	
C3	0.3689 (2)	0.4380 (4)	0.68582 (12)	0.0343 (5)	
C4	0.3783 (2)	0.2877 (3)	0.64026 (11)	0.0308 (5)	
C5	0.4551 (4)	0.6010 (4)	0.79203 (16)	0.0553 (8)	
H5A	0.5224	0.5753	0.8280	0.083*	
H5B	0.3659	0.6084	0.8123	0.083*	
H5C	0.4760	0.7195	0.7698	0.083*	
C6	0.3088 (3)	0.1409 (3)	0.53025 (12)	0.0360 (5)	
H6	0.3451	0.0203	0.5480	0.043*	
C7	0.4063 (2)	0.2217 (4)	0.47943 (12)	0.0371 (6)	
H7A	0.4223	0.1297	0.4428	0.045*	
H7B	0.4934	0.2472	0.5029	0.045*	
C8	0.3519 (3)	0.4009 (4)	0.44790 (13)	0.0387 (6)	
H8A	0.4163	0.4467	0.4138	0.046*	
H8B	0.3452	0.4964	0.4841	0.046*	
C9	0.2144 (3)	0.3769 (3)	0.41323 (12)	0.0337 (5)	
C10	0.1169 (2)	0.2822 (4)	0.46142 (13)	0.0359 (5)	
H10A	0.0340	0.2493	0.4354	0.043*	
H10B	0.0919	0.3715	0.4976	0.043*	
C11	0.1739 (3)	0.1065 (3)	0.49515 (14)	0.0400 (6)	
H11A	0.1095	0.0603	0.5292	0.048*	
H11B	0.1842	0.0088	0.4600	0.048*	
C12	0.1583 (3)	0.5624 (4)	0.38954 (16)	0.0529 (7)	
H12A	0.2230	0.6233	0.3597	0.079*	
H12B	0.1421	0.6415	0.4293	0.079*	
H12C	0.0737	0.5423	0.3643	0.079*	
N1	0.5510 (2)	0.3157 (3)	0.75037 (10)	0.0354 (5)	
N2	0.4744 (2)	0.1602 (3)	0.64825 (10)	0.0334 (4)	
N3	0.6521 (2)	0.0517 (4)	0.71160 (12)	0.0510 (6)	
H3A	0.6596	−0.0397	0.6820	0.061*	
H3B	0.7081	0.0588	0.7469	0.061*	
N4	0.2904 (2)	0.2682 (3)	0.58727 (10)	0.0355 (5)	
H4	0.2173	0.3370	0.5872	0.043*	
O1	0.23817 (18)	0.2603 (3)	0.35549 (9)	0.0420 (4)	
H1	0.1654	0.2413	0.3342	0.063*	
Br1	0.23506 (4)	0.62118 (5)	0.671478 (18)	0.06510 (16)	

### Atomic displacement parameters (Å^2^)

**Table:**

	*U*^11^	*U*^22^	*U*^33^	*U*^12^	*U*^13^	*U*^23^
C1	0.0334 (12)	0.0391 (13)	0.0292 (12)	0.0023 (10)	−0.0052 (10)	−0.0036 (10)
C2	0.0362 (13)	0.0361 (12)	0.0311 (12)	−0.0024 (10)	0.0010 (10)	−0.0052 (10)
C3	0.0341 (13)	0.0357 (12)	0.0330 (12)	0.0048 (10)	−0.0012 (10)	−0.0026 (10)
C4	0.0305 (12)	0.0357 (13)	0.0261 (11)	−0.0010 (9)	−0.0015 (9)	0.0012 (9)
C5	0.0625 (19)	0.0506 (17)	0.0523 (18)	0.0074 (14)	−0.0080 (15)	−0.0204 (14)
C6	0.0397 (14)	0.0341 (13)	0.0336 (13)	0.0029 (10)	−0.0131 (10)	−0.0028 (10)
C7	0.0305 (12)	0.0488 (15)	0.0318 (12)	0.0033 (10)	−0.0085 (10)	−0.0130 (11)
C8	0.0392 (14)	0.0455 (14)	0.0315 (12)	−0.0098 (11)	0.0002 (10)	−0.0048 (11)
C9	0.0378 (13)	0.0344 (12)	0.0289 (12)	0.0038 (10)	−0.0043 (10)	−0.0023 (10)
C10	0.0308 (12)	0.0419 (14)	0.0346 (12)	0.0000 (10)	−0.0085 (10)	0.0023 (11)
C11	0.0418 (14)	0.0376 (14)	0.0402 (14)	−0.0080 (11)	−0.0122 (11)	0.0056 (11)
C12	0.070 (2)	0.0410 (15)	0.0473 (17)	0.0115 (14)	0.0002 (15)	0.0079 (13)
N1	0.0342 (11)	0.0412 (11)	0.0306 (10)	0.0010 (9)	−0.0056 (8)	−0.0077 (9)
N2	0.0359 (11)	0.0374 (11)	0.0267 (10)	0.0035 (8)	−0.0071 (8)	−0.0059 (8)
N3	0.0541 (14)	0.0575 (14)	0.0405 (12)	0.0239 (12)	−0.0226 (11)	−0.0177 (11)
N4	0.0321 (10)	0.0446 (12)	0.0297 (10)	0.0073 (9)	−0.0075 (8)	−0.0030 (9)
O1	0.0410 (10)	0.0525 (11)	0.0319 (9)	0.0119 (8)	−0.0100 (7)	−0.0119 (8)
Br1	0.0679 (3)	0.0608 (2)	0.0659 (3)	0.03307 (16)	−0.01891 (17)	−0.01908 (15)

### Geometric parameters (Å, °)

**Table:**

C1—N3	1.335 (3)	C7—H7B	0.9900
C1—N2	1.340 (3)	C8—C9	1.526 (3)
C1—N1	1.349 (3)	C8—H8A	0.9900
C2—N1	1.352 (3)	C8—H8B	0.9900
C2—C3	1.366 (3)	C9—O1	1.430 (3)
C2—C5	1.493 (4)	C9—C12	1.514 (4)
C3—C4	1.405 (3)	C9—C10	1.525 (3)
C3—Br1	1.891 (2)	C10—C11	1.529 (3)
C4—N2	1.331 (3)	C10—H10A	0.9900
C4—N4	1.351 (3)	C10—H10B	0.9900
C5—H5A	0.9800	C11—H11A	0.9900
C5—H5B	0.9800	C11—H11B	0.9900
C5—H5C	0.9800	C12—H12A	0.9800
C6—N4	1.457 (3)	C12—H12B	0.9800
C6—C11	1.517 (3)	C12—H12C	0.9800
C6—C7	1.518 (4)	N3—H3A	0.8800
C6—H6	1.0000	N3—H3B	0.8800
C7—C8	1.523 (4)	N4—H4	0.8800
C7—H7A	0.9900	O1—H1	0.8400
			
N3—C1—N2	116.8 (2)	H8A—C8—H8B	107.8
N3—C1—N1	116.6 (2)	O1—C9—C12	109.9 (2)
N2—C1—N1	126.6 (2)	O1—C9—C10	110.1 (2)
N1—C2—C3	120.5 (2)	C12—C9—C10	110.3 (2)
N1—C2—C5	115.7 (2)	O1—C9—C8	104.89 (19)
C3—C2—C5	123.8 (2)	C12—C9—C8	111.0 (2)
C2—C3—C4	119.2 (2)	C10—C9—C8	110.5 (2)
C2—C3—Br1	121.01 (19)	C9—C10—C11	113.6 (2)
C4—C3—Br1	119.76 (17)	C9—C10—H10A	108.8
N2—C4—N4	118.2 (2)	C11—C10—H10A	108.8
N2—C4—C3	120.7 (2)	C9—C10—H10B	108.8
N4—C4—C3	121.1 (2)	C11—C10—H10B	108.8
C2—C5—H5A	109.5	H10A—C10—H10B	107.7
C2—C5—H5B	109.5	C6—C11—C10	112.2 (2)
H5A—C5—H5B	109.5	C6—C11—H11A	109.2
C2—C5—H5C	109.5	C10—C11—H11A	109.2
H5A—C5—H5C	109.5	C6—C11—H11B	109.2
H5B—C5—H5C	109.5	C10—C11—H11B	109.2
N4—C6—C11	109.1 (2)	H11A—C11—H11B	107.9
N4—C6—C7	110.6 (2)	C9—C12—H12A	109.5
C11—C6—C7	109.7 (2)	C9—C12—H12B	109.5
N4—C6—H6	109.1	H12A—C12—H12B	109.5
C11—C6—H6	109.1	C9—C12—H12C	109.5
C7—C6—H6	109.1	H12A—C12—H12C	109.5
C6—C7—C8	111.2 (2)	H12B—C12—H12C	109.5
C6—C7—H7A	109.4	C1—N1—C2	116.5 (2)
C8—C7—H7A	109.4	C4—N2—C1	116.4 (2)
C6—C7—H7B	109.4	C1—N3—H3A	120.0
C8—C7—H7B	109.4	C1—N3—H3B	120.0
H7A—C7—H7B	108.0	H3A—N3—H3B	120.0
C7—C8—C9	113.2 (2)	C4—N4—C6	124.3 (2)
C7—C8—H8A	108.9	C4—N4—H4	117.8
C9—C8—H8A	108.9	C6—N4—H4	117.8
C7—C8—H8B	108.9	C9—O1—H1	109.5
C9—C8—H8B	108.9		

### Hydrogen-bond geometry (Å, °)

**Table:**

*D*—H···*A*	*D*—H	H···*A*	*D*···*A*	*D*—H···*A*
N3—H3A···O1^i^	0.88	2.03	2.828 (3)	151
O1—H1···N1^ii^	0.84	2.02	2.803 (3)	155

Symmetry codes: (i) −*x*+1, −*y*, −*z*+1; (ii) *x*−1/2, −*y*+1/2, *z*−1/2.
